# Cholecystectomy in Patients with Liver Cirrhosis

**DOI:** 10.1155/2015/783823

**Published:** 2015-12-14

**Authors:** Jonas Strömberg, Folke Hammarqvist, Omid Sadr-Azodi, Gabriel Sandblom

**Affiliations:** ^1^Department of Surgery, Kalmar County Hospital, 39185 Kalmar, Sweden; ^2^Division of Surgery, CLINTEC, Karolinska University Hospital, Huddinge, Sweden; ^3^Upper Gastrointestinal Research, Department of Molecular Medicine and Surgery, Karolinska Institute, Stockholm, Sweden

## Abstract

*Background*. The aim of this population-based study was to describe characteristics of patients with liver cirrhosis undergoing cholecystectomy and evaluate the risk for perioperative and postoperative complications during the 30-day postoperative period. *Method*. All laparoscopic and open cholecystectomy procedures registered between 2006 and 2011 in the Swedish Registry for Gallstone Surgery and ERCP (GallRiks) were included. Patients with liver cirrhosis were identified by linking data to the Swedish National Patient Registry (NPR). *Results*. Of 62,488 patients undergoing cholecystectomy, 77 (0.12%) had cirrhosis, of which 29 patients (37.7%) had decompensated cirrhosis. Patients with cirrhosis were older and had more often gallstone complications at the time for surgery. Postoperative complications were registered in 13 (16.9%) patients with liver cirrhosis and in 5,738 (9.2%) patients in the noncirrhotic group (*P* < 0.05). Univariable analysis showed that patients with liver cirrhosis are more likely to receive postoperative blood transfusion (OR = 4.4, CI 1.08–18.0, *P* < 0.05) and antibiotic treatment >1 day (OR = 2.3, CI 1.11–4.84, *P* < 0.05) than noncirrhotic patients. *Conclusion*. Patients with cirrhosis undergoing cholecystectomy have a higher incidence of postoperative complications than patients without cirrhosis. However, cholecystectomy is safe and if presented with adequate indication, surgery should not be delayed due to fears of surgical complications.

## 1. Introduction

Gallstones are twice as common in patients with liver cirrhosis [1]. The incidence of gallstone disease is 9.5–29.4% compared to 5.2–12.8% in noncirrhotic patients [1, 2]. This predisposition has been attributed to several factors such as gallbladder dysmotility in the fibrous transformed liver, reduction in bile acidity, increased unconjugated bilirubin secretion, and increased intravascular hemolysis due to hypersplenism [3, 4]. The annual death rate from liver cirrhosis in Europe is around 170,000 [5]. The WHO mortality database reports that Hungary has the highest age-standardized death rate (103.3 per 100,000 men and 32.0 per 100,000 women) from liver cirrhosis in Europe [5]. In Sweden, the incidence rate is 15.3 per 100,000 inhabitants [6] and the mortality rate is one of the lowest in Europe (5.9 per 100,000 men and 2.7 per 100,000 women) [5].

Symptomatic gallstones in cirrhotic patients are associated with higher mortality and morbidity compared to the rest of the population [7]. However, surgical procedures in patients with a cirrhotic liver are considered a higher risk than in noncirrhotic patients. Therefore, the risk for developing complicated gallstone disease must be strictly weighed against the risk of surgery. Cholecystectomy is the most common surgical procedure in cirrhotic patients and known complications include perioperative bleeding, hepatic failure, kidney failure, postoperative infection, and impaired wound closure [8–10]. The severity of cirrhotic disease and the type of operation contribute to predicting operative risk and patient outcome [2].

In 1992, a consensus statement from the NIH considered liver cirrhosis to be a contraindication for laparoscopic cholecystectomy (LC) [11]. Since then, laparoscopy has evolved and today there are a number of publications advocating the safety of LC in selected cirrhotic patients, mainly Child-Pugh class A or B [12–16]. However, the clinical applicability of these results is uncertain with respect to the small population sizes. There are only a few larger studies on patients with liver cirrhosis undergoing cholecystectomy. In fact, only four retrospective [4, 17–19] and two prospective randomized studies [20, 21] have included more than fifty patients and report a wide spread of postoperative morbidity (6.6–47.3%) and mortality (0–4.3%). We therefore undertook a population-based study to assess the incidence and risk for perioperative and postoperative complications in cirrhotic patients within 30 days after cholecystectomy.

## 2. Methods

### 2.1. Study Design

In the present study the Swedish Registry for Gallstone Surgery and Endoscopic Retrograde Cholangiopancreatography (GallRiks) [22] and the Swedish National Patient Registry (NPR) were used [23]. All cholecystectomies registered in GallRiks between 2006 and 2011 were included and data were linked with the NPR to identify patients with liver cirrhosis. All cirrhotic patients had been diagnosed with liver cirrhosis prior to the time of surgery and were regarded as having decompensated cirrhosis if displaying symptoms of ascites, esophageal varices, or hepatic encephalopathy. Data on perioperative complications were extracted from GallRiks, and data regarding postoperative complications within 30 days after cholecystectomy were extracted from both GallRiks and/or the NPR. Patients were excluded if cholecystectomy was secondary to other procedures or if registration was incomplete for any of the variables studied.

### 2.2. Data Sources

*GallRiks* is an internet-based quality register for patients in Sweden that undergo cholecystectomy (laparoscopic/open) or Endoscopic Retrograde Cholangiopancreatography (ERCP) due to gallstone disease. The registry was founded in 2005 and since then has expanded to nationwide coverage. In fact, 99% of all hospitals in Sweden participate, and approximately 90% of all cholecystectomies performed in the country are registered in GallRiks [24]. The aim of the registry is to provide a continuous source of information for research and development regarding interventions on patients with gallstone disease. In addition, participating hospitals and surgeons are able to extract updated individual reports on complications and operative data from the registry. Patient characteristics, operation time, surgical indications, operative approach, and perioperative complications are reported on-line by the surgeon performing the cholecystectomy or ERCP. Medical records are reviewed for postoperative complications after 30 days by a local coordinator at each participating hospital. Registrations in GallRiks are continuously validated by independent observers. Medical records from each participating hospital are reviewed in a blinded audit every third year and compared to entries in the registry. Previous audits have shown a high validity with correct registration in 97.9% of all cases [22].

The* National Patient Registry* (*NPR*) has collected information and discharge diagnoses for all hospitalized patients in Sweden since 1964. The registry contains over 50 million discharges for the period 1964 to 2006. The NPR has full national coverage and includes data on all medical care in Sweden except primary care visits. Discharge diagnoses are coded by using the International Classification of Diseases (ICD-10) system. Currently over 99% of all discharges are covered and a recent validation showed correct registration in 85–95% of all cases [25].

### 2.3. Statistical Analyses

Patients with cirrhosis were compared to noncirrhotic patients regarding baseline characteristics and perioperative and postoperative complications using the Chi-Square test. Multivariable logistic regression analysis was performed to evaluate Odds Ratios (OR) on statistically significant variables. Missing data were detected for variables shown in Table 1, gender (*n* = 1), age (*n* = 2), ASA grade (*n* = 619), operation time (*n* = 1,239), operative approach (*n* = 1,354), and thromboembolism prophylaxis (*n* = 1,387), and in Table 2, pancreatitis (*n* = 2,209), bile duct obstruction (*n* = 2,216), cholangitis (*n* = 2,209), blood transfusion (*n* = 2,238), abscess (*n* = 2,210), bile leak (*n* = 2,213), and bleeding (*n* = 2,210). All individual missing data entries amounted to less than 4% of each variable and were therefore regarded as insignificant. IBM SPSS Statistics version 22.0 was used for all statistical analyses.

## 3. Results and Discussion

### 3.1. Results

A total of 62,488 patients were included of whom 77 patients (0.12%) had liver cirrhosis at the time of cholecystectomy. In this group, 29 patients (37.7%) had decompensated cirrhosis. Baseline characteristics (Table 1) show that gender was evenly distributed in the cirrhotic patient group but that women predominated (67%) in the noncirrhotic control group (*P* < 0.05). The majority of patients with cirrhosis undergoing cholecystectomy were older (64.9%) than noncirrhotics (40.2%; *P* < 0.001). In patients with liver cirrhosis, gallstone complication (cholecystitis, pancreatitis, or obstructive jaundice) indicated surgery more often than in the noncirrhotic patient group (50.6% and 37.3%, resp., *P* < 0.05). Operation time was increased (op. time > 90 min) in the cirrhosis group compared to noncirrhotics (*P* < 0.001). Open cholecystectomy was more frequently adopted in patients with liver cirrhosis (25.7%) than in the noncirrhosis group (9.0%; *P* < 0.001). Patients with liver cirrhosis had a higher conversion rate (13.5%) and received thromboembolism prophylaxis (TP) more often than noncirrhotic patients (*P* < 0.001).

Perioperative complications were seen in four patients (5.2%) with liver cirrhosis and in 2203 patients (3.5%) without cirrhosis (*P* = 0.43). Postoperative complications (Table 2) within 30 days after cholecystectomy were registered in 13 (16.9%) patients with liver cirrhosis compared to 5738 (9.2%) of patients without cirrhosis (*P* = 0.02). Furthermore, postoperative complications were seen in 5 patients (17.2%) and 8 patients (16.7%) in the decompensated and compensated cirrhosis group, respectively (*P* = 0.9).

Postoperative blood transfusion was given to two (2.9%) patients with liver cirrhosis and 402 (0.67%) patients in the noncirrhotic group (*P* < 0.05). Patients with liver cirrhosis had a significantly higher rate of postoperative infection (11.4%) that required antibiotic treatment >1 day than noncirrhotic patients (5.3%, *P* < 0.05). In a univariable subgroup analysis of patients with cirrhosis, the risk for postoperative complications with respect to different surgical approach was assessed. No significant difference was found when comparing open surgery (OR = 3.2, CI 0.906–11.06), conversion (OR = 1.2, CI 0.224–6.463), or laparoscopic surgery (OR = 0.353, CI 0.103–1.210).

In the group of patients with cirrhosis, univariable analysis predicted a significantly increased risk for postoperative blood transfusion (OR = 4.4, CI 1.08–18.0, *P* < 0.05) and postoperative infection requiring antibiotic treatment (OR = 2.3, CI 1.11–4.84, *P* < 0.05). Furthermore, the combined endpoint (any postoperative complication) was significantly increased in patients with liver cirrhosis (OR = 2.0, CI 1.11–3.65, *P* < 0.05). There was no mortality and none of the patients with liver cirrhosis developed postoperative liver failure within the first 30 days of the postoperative period.

### 3.2. Discussion

In the present population-based study we have combined two Swedish quality patient registers to investigate postoperative complications and outcome of patients with liver cirrhosis within 30 days after cholecystectomy. Perioperative complication rates were not found to significantly differ when comparing cirrhotic versus noncirrhotic patients undergoing cholecystectomy. However, we found that patients with cirrhosis had a greater incidence of postoperative complications. There was a higher incidence of patients needing postoperative blood transfusion (OR = 4.4, CI 1.08–18.0, *P* < 0.05), suggesting an increased bleeding tendency in this patient group. This increased risk for bleeding has previously been studied and attributed to a number of different factors. Patients with liver cirrhosis have a reduced capacity to produce coagulation factors [26]. In addition, portal hypertension generates thrombocytopenia due to hypersplenism and stimulates angiogenesis [9]. The neovascularization in the abdominal wall and around the liver hilum constitutes a technical challenge when performing cholecystectomy in cirrhotic patients [3]. In some cases, percutaneous cholecystostomy has been suggested for cholecystitis in patients with severe portal hypertension or with liver cirrhosis graded Child-Pugh C [19].

As well as being “naturally anticoagulated,” patients with cirrhosis also have low blood pressure, low concentrations of serum cholesterol, and a lower prevalence of acute myocardial infarction (AMI) [27]. Furthermore, contributing to the complexity of blood coagulation in liver cirrhotic patients, the anticoagulant proteins C and S are suppressed [26]. In a retrospective case-control study by Gulley et al., the medical records of 963 patients with cirrhosis were reviewed to establish the incidence of deep venous thrombosis (DVT) and pulmonary embolism (PE). The incidence of DVT/PE events in cirrhotic versus noncirrhotic patients was 1.87% and 0.98%, respectively. However, in multivariable analysis, liver cirrhosis was not found to be a risk factor for DVT/PE (OR = 0.86, *P* = 0.06) [26]. A recent review by Buresi et al. highlights the absence of evidence-based recommendations regarding administration of deep venous thromboembolism prophylaxis (TP) in patients with liver cirrhosis [28]. In this context and with consideration to the fact that TP is associated with increased risk for bleeding complications [29], it is notable that TP is given to 62.2% of all patients with liver cirrhosis undergoing cholecystectomy. This high rate could perhaps be explained by the fact that patients with cirrhosis were found to be older at the time for surgery in conjunction with the lack of consensus regarding TP in this patient group. In the present study, no DVT, PE, AMI, or stroke occurred within 30 days after cholecystectomy in patients with liver cirrhosis.

In the present study, OC was performed in 25.7% of cirrhotic and 9.0% of noncirrhotic patients undergoing cholecystectomy. This high rate of open surgical approach is interesting since it has previously been shown that LC has major advantages compared to open cholecystectomy in patients with liver cirrhosis [7]. However, there are some technical difficulties with performing laparoscopic cholecystectomy in patients with cirrhosis. The cirrhotic liver parenchyma is stiff from fibrous transformation and could interfere with the frequently used maneuver in LC where retraction of the gallbladder fundus is performed to expose the triangle of Calot [7]. In order to avoid this difficulty, some authors have proposed the insertion of an additional 5 mm trochar and a liver retractor [3, 18]. The pneumoperitoneum in LC has also been a concern due to presumed reduction in blood flow to the liver and kidney parenchyma [3, 7]. It has therefore been suggested that laparoscopic procedures in patients with liver cirrhosis are performed with a lower intra-abdominal pressure [3]. Finally, in dealing with the difficult liver bed and hilum in patients with portal hypertension, some authors have mandated the use of ultrasonic shears or performed laparoscopic subtotal cholecystectomy [18].

It is widely accepted that patients with liver cirrhosis are at higher risk of developing complications to surgical procedures. Therefore, a number of factors for preoperative optimization have been suggested. Vitamin K may be administered preoperatively, since malabsorption causes vitamin K deficiency that further exacerbates the inherent coagulopathy of the cirrhotic patient. Reduced levels of coagulation factors can be replaced by fresh frozen plasma or cryoprecipitate. Furthermore, the administration of blood platelets may be considered if the preoperative platelet count is less than 50.000/mL [9]. The portal hypertension seen in patients with liver cirrhosis may lead to the hepatorenal syndrome. This condition is caused by inadequate circulation to the kidneys due to splanchnic arterial dilatation [30]. The hepatorenal syndrome can be prevented by keeping patients well hydrated and in some cases may be reversed by the use of vasopressor agents [31]. Finally, ascites is common in patients with decompensated liver cirrhosis and increases the risk for wound dehiscence, intra-abdominal infection, and respiratory insufficiency [9]. Ascites development can be limited by the preoperative administration of diuretics or by laparocentesis.

It can be seen in Table 1 that patients undergoing cholecystectomy were older (50–70 years) in the cirrhosis group (64.9%) than in the noncirrhosis group (40.2%, *P* < 0.001). These results agree with those in a study by Yeh et al., where the median age for 226 cirrhotic patients undergoing LC was 57.7 years compared to 51.4 years in patients without cirrhosis [19]. One reason for cirrhotic patients being older when undergoing cholecystectomy could be that surgery is delayed due to the fear of increased surgical risk in this patient group. A postponement of intervention might also explain the higher frequency of gallstone complications at the time of surgery. When taking into consideration that emergency surgery is an independent risk factor for postoperative complications in this patient group [7], we believe that in many cases the decision to perform cholecystectomy should be taken at an earlier stage before gallstone complications occur.

One point of strength of the present study is that the results are population-based and that data are collected from the highly valid and carefully audited Swedish Registry for Gallstone Surgery and ERCP (GallRiks). Another is that we recover data on postoperative morbidity from both GallRiks and the Swedish National Patient Registry (NPR) (Table 2). In line with data from GallRiks, postoperative complications registered in the NPR were found not to differ significantly between the cirrhosis (9.1%) and noncirrhosis groups (3.1%). However, the present study has some limitations. There is no information on whether patients with cirrhosis were operated on in a specialized hepatobiliary center or by a general surgeon in a Swedish county hospital. Obviously, the level of specialized care could play a role in patient outcome. Furthermore, we do not have information regarding the severity of liver disease, as defined by the Child-Pugh scale, or if patients had been preoperatively optimized in any way.

## 4. Conclusions

Cirrhotic patients are at higher risk of developing postoperative infection (antibiotic requiring) and a higher number of patients are requiring postoperative blood transfusion, suggesting a bleeding tendency in this patient group. However, cholecystectomy should not be delayed and can be performed as a safe procedure in patients with compensated and decompensated liver cirrhosis.

## Figures and Tables

**Table 1:** Baseline characteristics.

	Number of data available	Cirrhosis *N* = 77 (%)	Noncirrhosis *N* = 62,411 (%)	*P* ^*∗*^
Gender	62487			<0.05
Male		38 (49.4)	20590 (33.0)	
Female		39 (50.6)	41820 (67.0)	
Age (years)	62486			<0.001
<50		10 (13.0)	29666 (47.5)	
50–70		50 (64.9)	25089 (40.2)	
>70		17 (22.1)	7654 (12.3)	
ASA grade	61869			<0.001
1		15 (20.0)	33666 (54.5)	
2		31 (41.3)	23678 (38.3)	
>2		29 (38.7)	4450 (7.2)	
Operative indication	62488			<0.05
Gallstone colic		38 (49.4)	39110 (62.7)	
Gallstone complication^1^		39 (50.6)	23301 (37.3)	
Operation time (min)	61249			<0.001
<90		23 (31.1)	30309 (49.5)	
>90		51 (68.9)	30863 (50.5)	
Operative approach	61134			<0.001
Laparoscopic		44 (59.5)	49093 (80.4)	
Open		19 (25.7)	5502 (9.0)	
Conversion		10 (13.5)	4778 (7.8)	
Thromboembolism prophylaxis	61101			<0.001
Yes		46 (62.2)	26171 (42.9)	
No		28 (37.8)	34856 (57.1)	

^1^Gallstone complication: cholecystitis, pancreatitis, or obstructive jaundice. ASA: American Society of Anesthesiologists. Lap: laparoscopic. ^*∗*^Pearsons Chi-Square test.

**Table 2:** Postoperative complications within 30 days after cholecystectomy^1^.

	Number of data available	Cirrhosis *N* = 77 (%)	Noncirrhosis *N* = 62,411 (%)	*P*
Gallstone complications				
Pancreatitis	60279	1 (1.3)	374 (0.6)	n.s
Bile duct obstruction	60272	1 (1.3)	589 (0.94)	n.s
Cholangitis	60279	0 (0)	129 (0.21)	n.s
Cardiovascular complications				
Acute myocardial infarct	62488	0 (0)	75 (0.12)	n.s
DVT	62488	0 (0)	36 (0.06)	n.s
PE	62488	0 (0)	25 (0.04)	n.s
Stroke	62488	0 (0)	92 (0.15)	n.s
Surgical complications				
Antibiotic compl.^2^	60251	8 (11.4)	3174 (5.3)	<0.05
Blood transfusion	60250	2 (2.9)	402 (0.67)	<0.05
Liver failure	62430	0 (0)	5 (0.01)	n.s
Wound infection	62488	1 (1.3)	153 (0.25)	n.s
Abscess	60278	2 (2.6)	834 (1.3)	n.s
Sepsis	62430	1 (1.3)	49 (0.07)	n.s
Bile leak	60275	0 (0)	804 (1.3)	n.s
Wound dehiscence	62488	0 (0)	15 (0.02)	n.s
Bleeding	60278	0 (0)	498 (0.83)	n.s
Perforation	62488	0 (0)	2	n.s
Reoperation	62488	0 (0)	34 (0.05)	n.s
Mortality	62488	0 (0)	127 (0.20)	n.s
Other complications	62488	3 (3.9)	2,099 (3.3)	n.s

^1^Postoperative complications from GallRiks and/or NPR. n.s = nonsignificant.

^2^Patients with postoperative infection requiring antibiotic treatment >1 day.
